# A Method for Energy Balance and Data Transmission Optimal Routing in Wireless Sensor Networks

**DOI:** 10.3390/s19133017

**Published:** 2019-07-09

**Authors:** Xuesong Liu, Jie Wu

**Affiliations:** 1 Department of Modern Physics, University of Science and Technology of China, Hefei 230026, China; 2 State Key Laboratory of Particle Detection and Electronics, University of Science and Technology of China, Hefei 230026, China

**Keywords:** wireless sensor network, energy balance, optimal routing, clustering routing protocol

## Abstract

Wireless sensor networks are widely used in many fields. Nodes in the network are typically powered by batteries. Because the energy consumption of wireless communication is related to the transmission distance, the energy consumption of nodes in different locations is different, resulting in uneven energy distribution of nodes. In some special applications, all nodes are required to work at the same time, and the uneven energy distribution makes the effective working time of the system subject to the node with the largest energy consumption. The commonly used clustering protocol can play a role in balancing energy consumption, but it does not achieve optimal energy consumption. This paper proposes to use the power supply line to connect the nodes to fully balance the energy. The connection scheme with the shortest power line length is also proposed. On the basis of energy balance, the method of transmitting data with the best hop count is proposed, which fully reduces the power consumption of the data transmission. The simulation results show that the proposed method can effectively reduce the energy consumption and prolong the lifetime of wireless sensor networks.

## 1. Introduction

**Figure 1:**
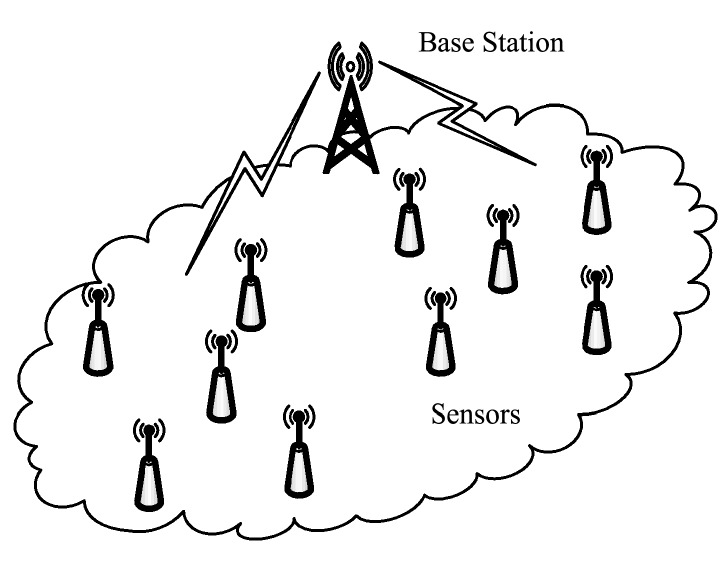
Application of wireless sensor networks.

Wireless sensor networks (WSNs) are widely used in industrial control [1,2,3], urban management, environmental monitoring and other fields [4,5,6]. In a wireless sensor network (WSN), a large number of sensor nodes collaboratively perceive, collect and process the information of the sensing object, and transmit the acquired information to the user terminal through wireless communication. Figure 1 shows a typical wireless sensor network. Sensor systems operating in the field are usually powered by batteries. Limited battery power limits the working hours of wireless sensor networks. Moreover, the power consumption of the nodes in the WSN is different, and some nodes exhaust energy in advance. In systems where data is required to be reliable and complete, or where as many nodes as possible are required to operate simultaneously, the node running out of energy in advance means that the system fails.

Structural health monitoring (SHM) systems are widely adopted to monitor the behavior of structures during forced vibration testing or natural excitation (e.g., earthquakes, winds, live loading). SHM aims at providing reliable data concerning the integrity of different kind of structures, in order to permit their further operational utilization or to impose their repair or retirement [7]. The data of multiple nodes in SHM is related. It is necessary to comprehensively analyze the data of each point to get the status information of the building. For example, monitoring of dams, bridges, railways, and coal mines, wireless sensor nodes are used for real-time monitoring and security alerts. Data anomalies in any area may indicate that danger is imminent, and security is guaranteed only when all monitoring nodes are working properly. In order to ensure that any small structural health hazards can be discovered during long-term monitoring, it is necessary to ensure that there are enough sensor nodes alive. If many nodes are exhausted, the system becomes unreliable. In a typical wireless sensor system, multiple sensors are used to repeatedly cover key locations. Redundant monitoring can increase system reliability, which means more nodes and higher maintenance costs. In the case of a certain number of nodes, it is necessary to make full use of the limited energy to improve the overall lifetime of the system and reduce the failure rate of the sensor nodes.

In the target tracking application of wireless sensor networks, a basic problem is target coverage. That is, given a set of targets and a WSN with some sensor nodes deployed over the monitoring field, target coverage problem is defined such that all the targets are continuously monitored or covered by at least one sensor node at any time [8,9]. When there are many nodes in the network running out of energy, the target is easily lost. To ensure that the target’s motion is captured, it is necessary to have as many nodes as possible to be alive. This also requires the full use of energy to extend the overall effective working time.

The most direct way to solve the shortage of power supply is to increase the capacity of batteries by forming batteries in parallel or in series. However, in the test, we found that the common lithium-ion batteries often have circuit break or short circuit fault. A battery pack consists of multiple batteries, and the failure of any one of the batteries will cause the failure of the battery pack, so the probability of failure of the battery pack is much higher than that of a single battery. For the system working in the field, it is inconvenient to replace batteries frequently, and the interruption of data acquisition caused by battery failure is intolerable. Balancing energy and prolonging system lifetime are hot issues in wireless sensor networks. This paper studies a scheme which can guarantee the system to work for a long time. Firstly, the energy waste problem in wireless sensor networks is analyzed, and then the principle of this scheme is given.

## 2. Analysis of Clustering Protocol in Wireless Sensor Networks

In the wireless sensor network application shown in Figure 1, the acquisition node can send data to the base station directly or in multi-hop mode. The energy consumption of nodes is related to the number and distance of data transmission. Nodes that are far away from the base station or have more forwarding tasks consume more energy than other nodes, which causes energy imbalance and makes some nodes exhaust energy ahead of time. A research hotspot in WSNs is to solve the problem of energy imbalance [10,11,12].

The common solution is to apply clustering routing protocol [13]. The basic idea of clustering protocol is to divide all acquisition nodes into several clusters. The cluster head is responsible for collecting the data in the cluster and sending it to the base station [14]. Because the cluster head takes on a lot of data transmission work, it consumes more energy than other nodes. The cluster head is re-elected periodically in the cluster, so that the work of sending data is distributed equally. Low-Energy Adaptive Clustering Hierarchy (LEACH) protocol is a typical clustering protocol in wireless sensor networks [15]. It elects cluster heads randomly and sends data to base stations in a single hop. The probability of each acquisition node being elected as cluster head is equal, and the energy consumption of each node in the cluster is the same. However, due to the different distances from each cluster to the central node, there are differences in energy consumption among different clusters, and the farther clusters deplete energy earlier. Many protocols have made some improvements to LEACH [16,17,18,19], mostly focusing on clustering, cluster head election, routing and other issues, such as Hybrid Energy-Efficient Distributed Clustering, (HEED) protocol [20]. Some studies use renewable energy to replenish energy in wireless sensor networks [21,22]. However, renewable energy sources are unstable and unevenly distributed and cannot be used as a reliable power source. Other studies have optimized data collection, data compression, and data transmission to reduce losses [23,24,25,26,27].

**Figure 2:**
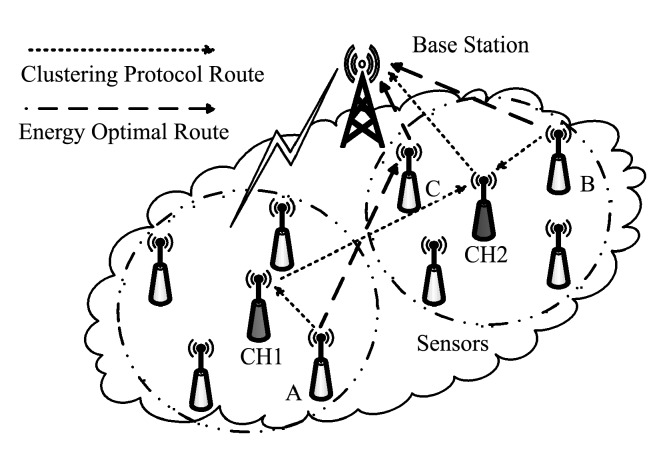
Route of clustering protocol in wireless sensor networks.

Clustering protocol improves the energy imbalance in wireless sensor networks. However, from the perspective of the overall energy consumption of the system, the energy balance between nodes is achieved at the cost of wasting some node energy. The application of clustering protocol in a wireless sensor network is shown in Figure 2. The nodes are divided into two clusters, the cluster heads are CH 1 and CH2. For Node A, under clustering protocols such as EEUC (the Energy-Efficient Uneven Clustering), its data is sent along the path of A-CH1-CH2-BS, and arrives at the base station after two hops. Obviously, considering the overall energy consumption of the system, the A-C-BS path of data transmission consumes the least overall energy. Similarly, in the clustering protocol, Node B belongs to CH2 cluster, and its data transmission path is B-CH2-BS. However, if Node B sends data directly to BS, it will save overall energy. It can be seen that clustering protocol inevitably increases the number of hops and the total transfer distance from the acquisition node to the base station, which results in the waste of the overall energy of the system.

Therefore, in wireless sensor networks, the goal of minimizing the overall energy consumption and balancing the energy of all nodes cannot be achieved at the same time, so the system lifetime cannot be maximized. In this paper, a power line connection scheme for acquisition nodes is proposed, which enables the energy between acquisition nodes to be transferred to each other, so that the energy can be balanced. The node chooses a path that minimizes the overall energy consumption to transmit data, thus saving the most data transmission energy.

## 3. Energy Consumption Model for Sensor Networks

### 3.1. Energy Consumption Model for Data Transmission

**Figure 3:**
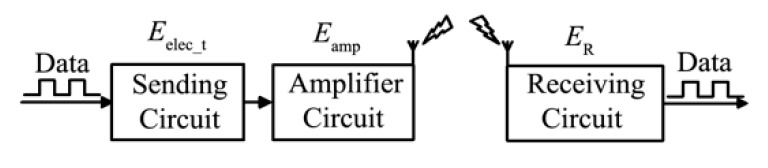
Radio energy consumption model.

We adopt the same radio energy consumption model as in [1], as shown in Figure 3.

The energy consumption in the process of data transmission consists of the following three parts.

1. The energy consumption *E_T_*(*d, m*) for sending *m* bit data to the node with a distance of *d* meters consists of two parts: sending circuit loss *E*_elec_t_ and amplifier circuit loss *E*_amp_.
  (1) $$\begin{array}{l} E_{T}\left(d,m\right)=E_{\mathrm{elec}\_t}+E_{\mathrm{amp}}\\ =\{\begin{array}{c} m\times E_{\mathrm{elec}}+m\times \varepsilon_{\mathrm{fs}}\times d^{2},(d<d_{0})\\ m\times E_{\mathrm{elec}}+m\times \varepsilon_{\mathrm{amp}}\times d^{4},\left(d\geq d_{0}\right) \end{array} \end{array}$$
  In the formula, *E*_elec_ denotes the data energy consumption of transmitting a unit bit, *ε*_fs_ denotes the data energy consumption of a unit bit in free space mode, *ε*_amp_ denotes the data energy consumption of a unit bit in multi-path attenuation mode, and $d_{0}=\sqrt{\varepsilon_{\mathrm{fs}}/\varepsilon_{\mathrm{amp}}}$ is the critical value for dividing the spatial model.
2. The energy consumption of receiving node receiving m bit data is *E*_R_(*m*).
  (2) $$E_{R}\left(m\right)=m\times E_{\mathrm{elec}},$$
3. The energy consumption of data transfer node fusing m bit data is *E*_DA_(*m*).
  (3) $$E_{\mathrm{DA}}\left(m\right)=m\times E_{\mathrm{da}},$$
  *E*_da_ is the energy required to fuse unit bit data.

### 3.2. Energy Consumption Model for Power Transfer

**Figure 4:**
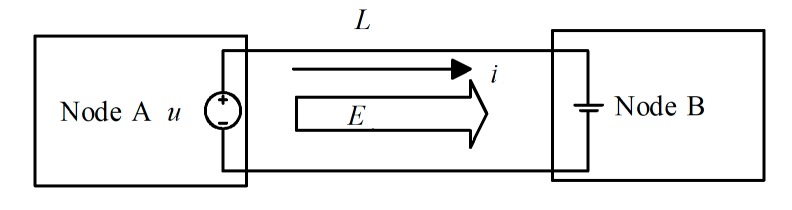
Energy transfer model.

Line loss is the heat loss caused by the current on the resistance when it flows through the cable. For example, *E* (*Joule*) energy is transferred from Node A to Node B, and the distance between the two nodes is *L* (*meter*)*,* as shown in Figure 4.

Suppose that the voltage on the power line is *u* (*V*), the current is *i* (*A*), and the resistance of the line is ***ρ*** (Ω∙m^−1^). The power of energy transfer is *P = u* × *i*, and the energy transfer time of *E* (*Joule*) by constant current is as follows

(4) $$t=\frac{E}{P}=\frac{E}{u\times i},$$

The total resistance of the power line is *R*

(5) R=2Lρ,

The energy loss is calculated as

(6) $$E_{\mathrm{loss}}=i^{2}Rt=\frac{2\rho i}{u}\times L\times E,$$

It can be seen that if energy is transferred by constant current and voltage, line loss is proportional to the transfer energy. For example, suppose the node operates at 5 V and the adjacent section delivers power at 5 mA, providing 25 mW of power. If the cable resistivity is 1 Ω∙m^−1^, and the two nodes are connected with a 10 m power line. The line loss of the transmitted energy is *E*_loss_ = 2%*E*. In practical applications, the voltage and current of the power transmission are selected according to the operating voltage and power consumption of the node. In any case, the energy loss caused by line loss is very limited.

## 4. Power Line Connection Scheme for Wireless Sensor Networks

### 4.1. Shortest Path of Connecting Nodes

The fundamental reason the energy in wireless sensor networks cannot be fully utilized is that the energy consumption of each node is different. We propose to connect all nodes with power lines, so that the energy between nodes can be transmitted to each other, so as to achieve energy balance.

**Figure 5:**
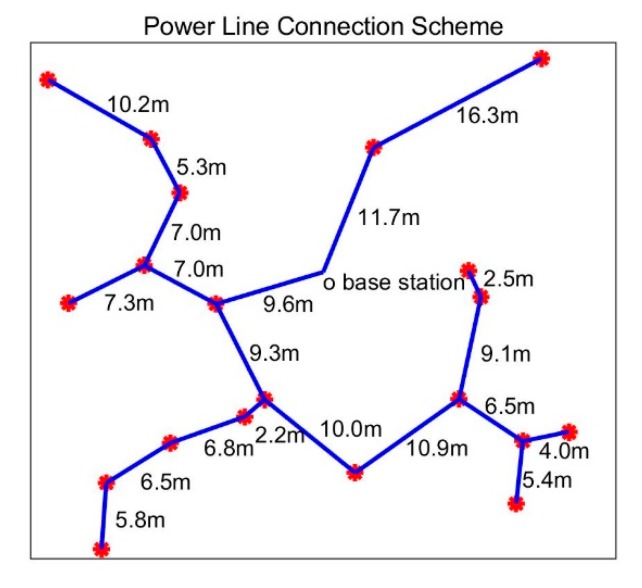
Power line connection scheme.

After the sensor nodes are deployed in a certain location, a shortest path can be found to connect all the nodes. A sensor network can be regarded as a weighted undirected graph, a node can be regarded as a vertex in the weighted undirected graph, and a connection between any two nodes can be regarded as an edge connecting two vertices, and its weight value is the length of the power line. The minimum weight spanning tree T of the weighted undirected graph can be found, and the sum of its weights is the smallest. Connecting power cables according to the topology of tree T is the shortest way to use cables. Figure 5 depicts a simple sensor network with 20 nodes randomly distributed. According to the distance information between two nodes, we can construct a minimum spanning tree based on the base station, and then connect the power line. The actual power line connection scheme is shown in the figure.

In the connection method in which the minimum spanning tree is applied, when a certain number of nodes are evenly distributed in a mesh shape, the total length of the cable required is the longest. In this case, the length of the cable connecting each node to the others is equal to the node spacing L (meter), so the average cable cost per node is W

(7) $$W=L\times P_{cable}$$

In the formula, *P_cable_* is the price per meter of the power cable. According to this formula, the system cable cost can be roughly estimated.

### 4.2. Estimation of Power Transmission Loss

In a sensor network with N nodes, a total of N−1 power lines need to be connected. As long as the length of each power line and the power transmitted through it are calculated separately, the overall power transmission loss of the system can be calculated according to Formula (6).

**Figure 6:**
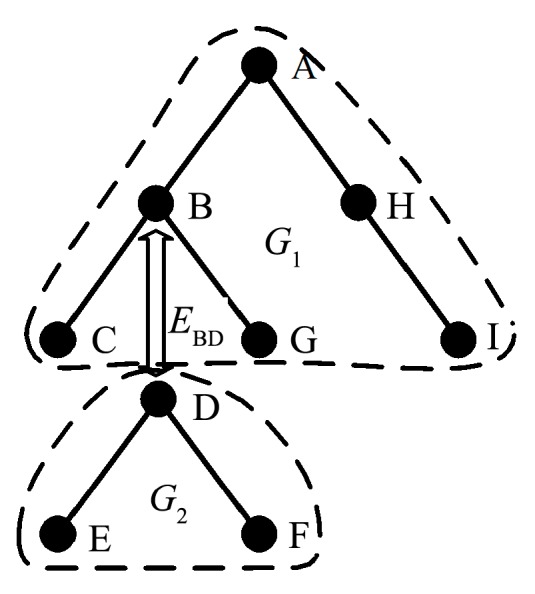
Minimum spanning tree topological connection and energy transfer.

Figure 6 shows the power line connection structure generated by a network of several nodes. To calculate the energy of electric energy transmission, the power line between nodes B and D in the figure is taken as an example. It divides all nodes into two groups: group $G_{1}=\left\{A,B,C,G,H,I\right\}$ near the tree root and group $G_{2}=\left\{D,E,F\right\}$ far from the tree root. The energy transmitted through the power cord is

$$E_{\mathrm{BD}}=\left|\sum \left(E_{i}-\bar{E}\right)\right|,i\in G_{1},$$

$\bar{E}$ is the average energy of all acquisition nodes.

For each node *N*, there is a unique precursor node *N*_pre_ in the minimum spanning tree. The length of the power line between the two nodes is *l_N_*. It divides all nodes into two groups, *G*_1_ and *G*_2_. *G*_2_ is a search tree with node *N* as its root, which is denoted as *T*(*N*). The transmission energy on this line is *E_N_*.

$$E_{N}=\left|\sum \left(E_{j}-\bar{E}\right)\right|\;,j\in T\left(N\right),$$

According to Formula (6), the loss on this power line are as follows

(8) $$E_{\mathrm{loss}}=\frac{2\rho i}{u}\times l_{N}\times \left|\sum \left(E_{j}-\bar{E}\right)\right|\;,j\in T\left(N\right),$$

The overall system loss is

(9) $$E_{\mathrm{loss}}=\sum E_{\mathrm{loss}}\left(j\right)$$

Formula (9) is used to estimate the loss caused by energy transmission over power lines.

Battery-powered station energy is represented by voltage, while high voltage means more energy. In a system where energy can be transmitted to each other, the node whose voltage is higher than the average output energy and the node whose voltage is lower than the average receives energy. In the application of the scheme, the average voltage should be set to the acquisition node. If the local battery voltage of the node is higher than the average value, the node outputs the local battery energy to the power line, otherwise, the node prefers to work with the energy provided by the power line, and saves part of the energy to the local battery.

### 4.3. Optimal Data Transmission Path Algorithms

In the scheme of power line connection, all nodes are fully balanced in energy, and each node can fully consider the overall energy optimization when selecting a data transmission path. A node farther from the base station, its data is transmitted to the base station in multiple hops. For a certain transmission distance, when the number of hops is too small, the distance of each hop is relatively long. Since energy consumption is proportional to the square of the distance, energy consumption is large. On the contrary, when there are too many hops, although the distance of each hop is close, and the energy consumption of one hop is small, the loss of additional receiving, transmitting, and merging data of the transit node increases. Therefore, choosing the right number of hops can save the energy of sending data. A node that is *d* meters away from the base station transmits 1 bit of data, and the data is transmitted to the base station through *n* hops. The total energy consumed in this process is $E_{\mathrm{bit}}\left(d,n\right)$.

(10) $$\begin{array}{c} E_{\mathrm{bit}}\left(d,n\right)=n\times E_{T}+\left(n-1\right)\times \left(E_{R}+E_{\mathrm{DA}}\right)\\ =\{\begin{array}{c} \begin{array}{l} n\times \left(E_{\mathrm{elec}}+\varepsilon_{\mathrm{fs}}\times {\left(\frac{d}{n}\right)}^{2}\right)+\\ \left(n-1\right)\times \left(E_{\mathrm{elec}}+E_{\mathrm{da}}\right),\frac{d}{n}<d_{0} \end{array}\\ \begin{array}{l} n\times \left(E_{\mathrm{elec}}+\varepsilon_{\mathrm{amp}}\times {\left(\frac{d}{n}\right)}^{4}\right)+\\ \left(n-1\right)\times \left(E_{\mathrm{elec}}+E_{\mathrm{da}}\right),\frac{d}{n}\geq d_{0} \end{array} \end{array} \end{array},$$

We can always find the right number of hops to make $E_{\mathrm{bit}}\left(d,n\right)$ the smallest. We refer to some parameters in [1].

$$E_{\mathrm{elec}}=50,\;\varepsilon_{\mathrm{fs}}=10,\varepsilon_{\mathrm{amp}}=0.0013,E_{\mathrm{da}}=5,d_{0}=87$$

**Figure 7:**
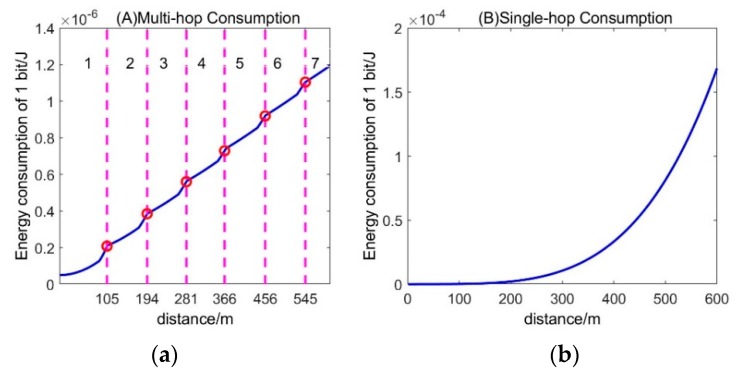
Minimum energy consumption and number of hops (**a**) and single hop energy consumption (**b**) for different distances.

Figure 7a shows the minimum energy required to transmit a single bit at different distances. The numbers indicated in the different intervals in the figure are the optimal number of hops that minimize the energy within this interval. Obviously, as the distance increases, the optimal hop count gradually increases. Overall, the energy consumption and distance through multi-hop transmission are approximately linear. When the distance is less than 105 m, the number of hops that minimize energy consumption is one. When the distance is greater than 105 m, the optimal hop count is increased by one for every *d*_0_ meter increase. That is to say, on the most energy-efficient communication path from one node to the base station, the distance between the two hops is within *d*_0_. Figure 7b shows the energy consumption for transmitting a single bit with a single hop. Comparing the energy consumption of single-hop and multi-hop, it can be concluded that as the distance increases, transmitting data according to the optimal hop count can significantly reduce energy consumption.

The base station can aggregate the location information of all nodes and calculate the distance from each node to the base station. According to Figure 7, the optimal hop count of each node and the distance of each hop can be determined, thereby planning the optimal transmission path of this node. The base station broadcasts the routing information to all nodes, and does not need to update the routing table during the subsequent work.

## 5. Simulation

In order to verify whether the power line connection scheme can effectively improve the system lifetime, simulation was performed with MATLAB. For clustering protocols such as LEACH and EEUC, the simulation is divided into three phases, the establishment phase, the cluster head election phase, and the data transmission phase. For our proposed power line connection scheme, no clustering is required. According to the method proposed above, the routing table is calculated at the beginning, and then the data is transmitted according to the routing information until the node energy is exhausted. Referring to Reference [8], the process by which all nodes collect and transmit a set of data packets is called a “round”. After completing a round of data acquisition and transmission, calculate the energy consumption of each node and the loss of energy transmission, and then continue to the next round. We assume that the cluster head is deployed in the center of the square area and does not compress the data. If the node is exhausted, it is removed from the system. The round represents the lifetime of the node. Some parameters used in the simulation are shown in Table 1. These parameters are set in the same way as Reference [11,13,15,16,17,18,19,20,28,29], and these parameters are also used in many WSN research articles. It is also convenient to compare different protocols with the same parameters.

The LEACH protocol is the most widely used clustering protocol, and many protocols are referenced to the LEACH protocol. CH-leach is an energy-efficient clustering protocol that uses the k-means method to select cluster heads [29]. The Energy-Efficient Uneven Clustering (EEUC) routing protocol uses uneven clustering, which makes the cluster size closer to the base station smaller and reduces the energy consumption in the cluster to reserve more energy to forward the data of the far cluster [28]. Simulation is carried out to compare the effect of our proposed scheme and these three protocols.

### 5.1. Simulation of Relationship between Node Density and Lifetime

**Figure 8:**
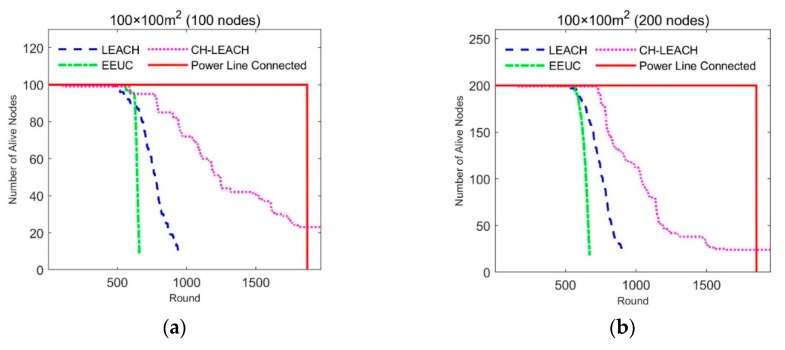

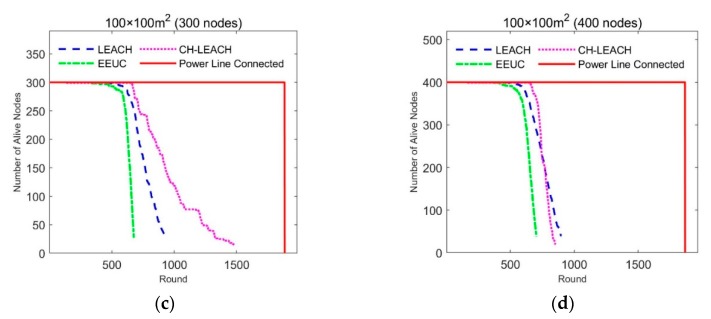
Simulation result, Relationship between node density and lifetime: (**a**) 100 × 100 m^2^ (100 nodes), low density area; (**b**) 100 × 100 m^2^ (200 nodes), middle density area; (**c**) 100 × 100 m^2^ (300 nodes), high density area; (**d**) 100 × 100 m^2^ (400 nodes), very high density area.

The density of nodes affects the effect of energy balance. We simulated the relationship between node density and system lifetime by simulating different number of nodes in the same size region. The simulation results are shown in Figure 8.

**Figure 9:**
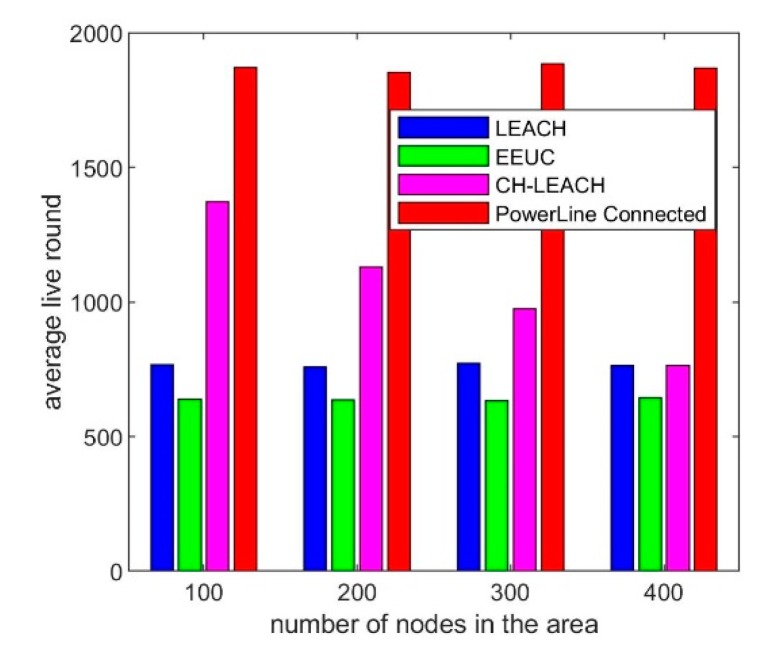
Simulation result, Network lifetime comparison at different node density.

The abscissa of each of the graphs in Figure 8 is round, representing the lifetime of the node. The ordinate is the number of alive nodes at each round. The simulation is stopped when the number of surviving nodes is less than 10%. The lifetimes of LEACH and EEUC vary little with node density, while the effect of CH-leach deteriorates as node density increases. In our proposed approach, all nodes work together until the energy is exhausted, and all nodes have the same lifetime and are much longer than other protocols. Figure 9 shows the comparison of the average lifetime of all nodes.

From the simulation results in the above figure, it can be seen that the lifetime of our scheme is longer than other protocols under different node densities. The average lifetime is 2.4, 2.9, and 1.8 times that of LEACH, EEUC, and CH-leach, respectively.

### 5.2. Simulation of Relationship between Area Size and Lifetime

**Figure 10:**
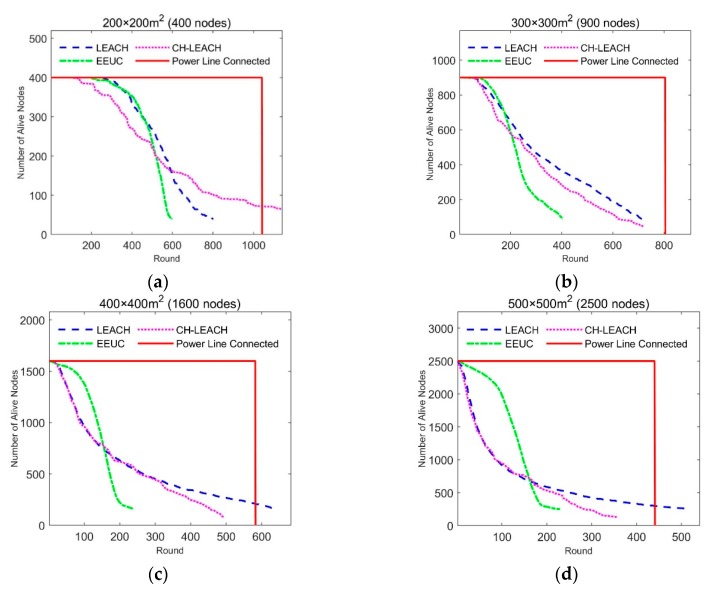
Simulation result, network lifetime comparison at different area sizes: (**a**) small area size, (**b**) middle area size, (**c**) large area size, (**d**) very large area size.

When the WSN system is deployed in different sizes, the energy consumption varies greatly. We chose a node density of 1 nodes/100 m^2^ and then simulate the case where the side length is 200, 300, 400, and 500 m. The simulation results are shown in Figure 10.

**Figure 11:**
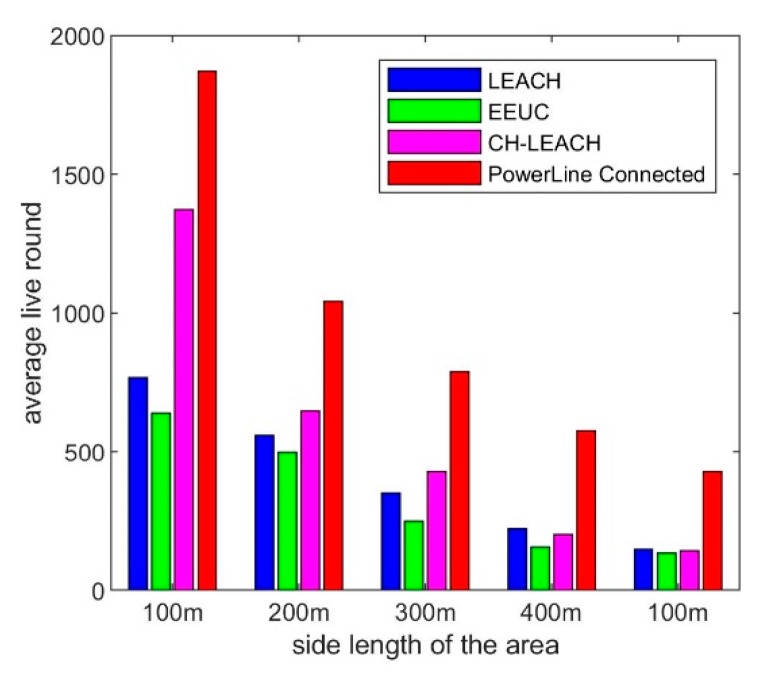
Simulation result, relationship between area size and lifetime.

At different sizes, the trend of the EEUC protocol is basically the same, that is, the node death is slow at the beginning, and then most of the nodes die quickly. When the area is large, the nodes at the beginning of LEACH and CH-leach will die quickly, and the remaining few nodes in the later stage can work for a long time. In our proposed solution, the system lifetime is longer than the other three options. For example, in the case of 400 m × 400 m and 1600 nodes, most of the nodes use up energy in about 150 rounds under the EEUC protocol. The Power Line Connected scheme can fully balance the energy between nodes, and all nodes can work about 580 rounds. Figure 11 is a comparison of lifetime in different area sizes.

From the comparison results in the above figure, in general, as the size of the area increases, the lifetime of these four schemes decreases. On average, the lifetime of our proposed solution is 2.3, 2.8, and 1.7 times that of LEACH, EEUC, and CH-leach, respectively.

### 5.3. Comparison of Energy Consumption with EEUC Protocol

**Figure 12:**
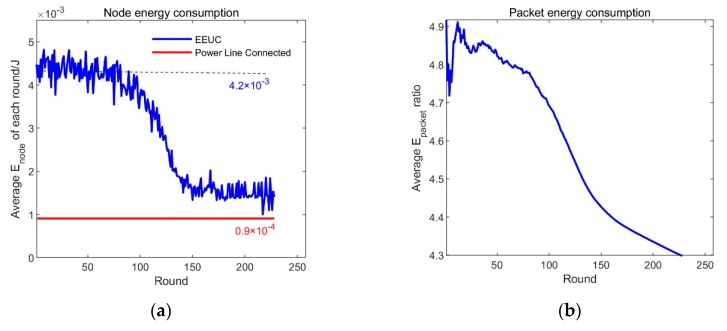
Node energy consumption and packet energy consumption (**a**) Node average energy consumption for each round, (**b**) Packet average energy consumption ratio.

Corresponding to the simulation result in Figure 11, Figure 12a shows the average energy consumption of the surviving nodes in each round $E_{\mathrm{NODE}}$.

$$E_{\mathrm{NODE}}=\frac{E_{\mathrm{ROUND}}}{N_{\mathrm{ALIVE}}},$$

where $E_{\mathrm{ROUND}}$ is the sum of energy consumption of all nodes in the round, $N_{\mathrm{ALIVE}}$ is the number of nodes alive in the round. In the whole working process of the system with power line connection, no node exhausts energy in advance and the communication route is unchanged, and the total energy consumption per round is also unchanged. The simulation results show that the average energy consumption of the nodes in each round is always $9.1\times 10^{-4}\;J$. Under the EEUC protocol, most nodes can survive for 100 rounds, and the average energy consumption in each round is about $4.2\times 10^{-3}\;J$. As a result, the power line connection scheme can reduce the node average energy consumption by about 78%.

According to the total energy consumed by the system and the total number of transmitted packets, the average energy consumed by each packet can be calculated.

$$E_{\mathrm{PACKET}}=\frac{E_{\mathrm{TOTAL}}}{N_{\mathrm{PACKET}}},$$

where $E_{\mathrm{TOTAL}}\;$ is the sum of the energy consumed by all nodes from the beginning of the work to the current round and $N_{\mathrm{PACKET}}$ is the total number of data packets transmitted by the system. Figure 10b shows the ratio of the average energy consumed per packet under the application of the power line connection scheme and the application of the EEUC protocol. In the EEUC scheme, when all nodes are dead, the energy consumption ratio is close to 4.3:1, which means that the power line connection scheme can transmit data with less energy consumption.

Based on the simulation results of Figure 12, the system with the power line connection reduces the average transmission power consumption by 78%, and the overall survival time can be increased by more than four times.

## 6. Analysis and Conclusion

The clustering algorithm commonly used in wireless sensor networks balances network energy and extends system operating time. However, intra-cluster communication and data forwarding between cluster heads cannot use the overall energy optimal path, which makes the energy of the whole system waste a lot. The power line connection method proposed in this paper fully balances the energy between nodes, so that all nodes can transmit data with the energy optimal path, saving the overall energy and significantly improving the system lifetime.

Although the power cable is added to the system, it is essentially different from the traditional wired data transmission system. In the wired data transmission system, the communication cable used for data transmission is very strict. To ensure stable and reliable communication, the cable impedance is required to be uniform and must be able to resist signal interference. In larger sensor systems, the large number of communication cables required for wired transmission makes the system expensive and difficult to deploy. In the form of wired communication, data can only be transmitted between nodes connecting cables, reducing communication flexibility. The power line is only the DC power supply between the connected nodes, and does not require the cable to have communication capability, the cost is low, and the deployment is relatively easy. In a complex field environment, cable disconnection may occur. If wireless communication is used, system data transmission will not be affected. A small number of power lines are disconnected to divide the entire connected system into several small connected systems, and the energy in each small system is still balanced.

It can be seen from the simulation results that the power conversion scheme of connecting the sensor nodes with the power line extends the working time of the wireless sensor network by more than four times. Most importantly, our proposal makes all nodes work longer. This is critical to systems that work together as a whole, ensuring that data is reliable and complete. In addition, in IoT (Internet of Things) applications, the energy of sensor nodes is the key to system performance [30,31]. Our proposal ensures that there is a continuous supply of power to key nodes in the IoT application, keeping the IoT system in high performance. Many wireless sensor networks collect renewable energy sources which are unevenly distributed. For example, solar energy, only nodes with sufficient light can collect enough energy, and the nodes placed in the shadows have no energy supplement. The scheme of connecting the power line can transfer energy from the sun-irradiated area to the shaded area, making full use of the energy collected by all nodes and making better use of renewable energy to extend the lifetime of the system.

Of course, the price paid is that a power line is connected between the nodes. This undoubtedly increases the system cost, which is mainly the cable cost and installation cost. Regarding the cable cost, a rough value can be calculated according to Equation (7). Power cables are inexpensive, but sensor prices vary widely. The ratio of the power cable cost to the total cost of the original wireless system is *r*

(11) $$r=\frac{W}{P_{sensor}}=\frac{L\times P_{cable}}{P_{sensor}},$$

In the Formula (11), *P_sensor_* is the price of a single sensor node. For some expensive sensors, *r* is small; for low-cost sensors, this ratio is considerable. For example, if a sensor system deploys 400 sensor nodes in a 100 m × 100 m area, then *L* = 5 m. We assume that the price of the power cable is about 1$ per meter, and the price of the sensor node is 100$ per unit. According to Equation (11), the cost of the power line connection scheme is increased by 5%. According to the simulation results of Figure 9, the system lifetime can be extended by about 2.3 times in this situation. Costs should be calculated based on cable and sensor prices in different applications. Regarding the installation cost, it depends largely on the environment in which the sensor system is deployed. Sensors and cables are easy to install in flat and open areas, and labor and time costs are small, while it is difficult and costly to deploy in mountains or tall buildings. Taking the application of a seismic exploration scenario as an example, a seismic acquisition system with 3000 channels has a cost of 80%–85% of the cost of a wireless system, and in a 12,500-channel seismic acquisition system with a higher density, the data is 50%–60% [32]. Of course, in different applications, the ratio of the installation cost of the power line to the total cost is also different, and the user can make a professional evaluation according to the actual situation. In our proposal, the cables are installed only once during system deployment and can be used throughout the lifetime of the system. Moreover, the fact that few nodes exhaust energy in advance means that the number of nodes can be reduced and the cost can be saved. In some application scenarios, it is cost-effective to obtain longer lifetime at the cost of connecting the power line. This method can effectively improve working hours in wireless systems of different scales, and may provide reference for other similar systems.

## Figures and Tables

**Table 1:** Simulation parameters.

Parameter	Value
Initial energy / J	0.5
Packet length / bit	4000
Energy consumption of transmitting and receiving circuits / nJ∙bit^−1^	50
*ε*_fs_ / pJ∙bit^−1^∙m^−2^	10
*ε*_amp_ / pJ∙bit^−1^∙m^−2^	0.0013
*E*_da_ / nJ∙bit^−1^	5
Energy transfer voltage (*u*) / V	5
Energy transfer current (*i*) / mA	10
Power line resistance (*ρ*) / Ω∙m^−1^	1
